# Supplementary material for: Mechanochemical feedback between confinement and actin crosslinking drives the shape dynamics of liquid-like droplets
Source: Nat Commun. 2026 Feb 23;17:3068. doi: 10.1038/s41467-026-69803-4 (PMC13039813; doi:10.1038/s41467-026-69803-4)
Supplement: Supplementary file 1 — Supplementary Information [file 41467_2026_69803_MOESM1_ESM.pdf]

Supplementary Information for

Mechanochemical feedback between confinement and actin crosslinking drives the shape dynamics of liquid-like droplets

Daniel Mansour[1], Dominique Jordan[2], Caleb Walker[2], Aravind Chandrasekaran[1], Christopher T. Lee[3], Kristin Graham[2], Jeanne Stachowiak[2,4]*, Padmini Rangamani[1,5]*

[1] *Department of Mechanical and Aerospace Engineering, University of California San Diego, La Jolla, CA, United States.*
[2] *Biomedical Engineering, The University of Texas at Austin, Austin, TX, United States.*
[3] *Department of Molecular Biology, University of California San Diego, La Jolla, CA, United States.*
[4] *Chemical Engineering, The University of Texas at Austin, Austin, TX, United States.*
[5] *Department of Pharmacology, University of California San Diego School of Medicine, La Jolla, CA, United States.*

**To whom correspondence should be addressed: prangamani@ucsd.edu, jcstach@austin.utexas.edu*

Supplementary Tables

Supplementary Table 1: Table of universal parameters used in the Cytosim model

Parameter	Value	Notes/Reference
Total time (T_{sim})	600 s	
Time step	0.002 s	
Droplet viscosity	0.5 pN s/μm^2	500x water;<br>Chosen based on protein condensate viscosities[1].
**Boundary**		
Initial Shape	Spherical, constrained to a deformable ellipsoid unless stated otherwise.	Rigid Ellipsoidal in Fig. 8A<br>Rigid Spherical in Supplementary Fig. 2
Effective Viscosity: $\mu_{\mathrm{effective}}$	100 pN s/μm unless stated otherwise.	Varied in Fig. 7, Supplementary Fig. 4, and Supplementary Fig. 7 to show that this parameter does not affect droplet deformation timescales in our study
Radius	1 μm unless stated otherwise.	Varied in power law simulations.
Boundary repulsion stiffness	200 pN/μm for actin filaments;<br>100 pN/μm for crosslinking molecules	This specifies the spring stiffness that acts on the discretized points of each actin filament and crosslinking molecule if the point lies outside the specified boundary. The force on each point is dependent on the distance that it lies beyond the confines of the boundary. Additionally, for actin filaments, these forces are used to calculate the point forces acting on the boundary that drive droplet deformation[2].
**Actin filaments**		
Segmentation length L_{seg}	0.1 μm (100 nm)	
Maximum length	2πR μm	
Polymerization rate k_{grow}	0.0103 μm/s for diameter 2 μm<br>General formula = $\frac{(2\pi R - L_{\mathrm{fil}}^{0})}{T_{\mathrm{sim}}}$	Only plus (+) end extension is allowed. This rate is calculated by assuming a final filament length of 2π μm at 600 s.

Brownian ratchet force for polymerization	10 pN	[3]
Actin flexural rigidity k_{bend}	0.075 pN μm^2	[4]
Actin steric repulsion k_{steric}	Radius 3.5 nm<br>Stiffness 1.0 pN/μm	Chosen to ensure the observation of ring structures within the kinetic parameters used in this study, as determined from a previous study[5].
**Tetravalent crosslinkers (VASP)**		
Radius	30 nm	
Diffusion rate	10 μm^2/s	
Concentration of tetramers	0.40 μM [1000 tetramers when Radius = 1 μm]	The number of simulated tetramers is scaled to maintain the equivalent concentration with varied droplet size.
Actin-binding rate<br>(Ring Kinetics)	10.0 (1/s)	Determined from VASP simulations.
Actin-binding rate<br>(Shell Kinetics)	0.1 (1/s)	Determined from VASP simulations.
Actin-binding distance	30 nm	
Actin-binding valency	4	Each spherical molecule approximates a VASP tetramer.
Zero-force actin-unbinding rate<br>(Ring and Shell Kinetics)	1.0 (1/s)	Determined from VASP simulations.
Actin-unbinding force	10 pN	Typical values for passive crosslinkers[6].
VASP steric repulsion	Radius 30 nm<br>Stiffness 10 pN/μm	Chosen to ensure the observation of ring structures within the kinetic parameters used in this study, as determined from a previous study on tetramers[5].
**Monovalent actin binders that interact with each other multivalently (mini-Lpd monomers)**		
Radius	30 nm	
Diffusion rate	10 μm^2/s	

Concentration of monomers	0.79 µM [2000 monomers when radius = 1 µm]	The number of simulated monomers is scaled to maintain the equivalent concentration with varied droplet size.
Actin-binding rate	10.0 (1/s)	Determined from VASP simulations.
Actin-binding distance	30 nm	
Actin-binding valency	1	Each spherical molecule approximates a mini-Lpd monomer.
Zero-force actin-unbinding rate	1.0 (1/s)	Determined from VASP and mini-Lpd simulations.
Actin-unbinding force	10 pN	Typical values for passive crosslinkers[6].
mini-Lpd monomer-actin steric repulsion	Radius R_{solid} = 30 nm<br>Stiffness 10 pN/µm	Chosen to ensure the observation of ring structures within the kinetic parameters used in this study, as determined from a previous study on tetramers and mini-Lpd monomers[5,7].
mini-Lpd monomer-monomer binding distance	90 nm ($3R_{solid}$)	This is the distance between the binding site on solid A and the center of solid B. So, if two solids are in contact, depending on the position of the binding site, this distance can scale between R_{solid} and $3R_{solid}$, where R_{solid} is the radius of the solid.
Monomer-monomer binding valency	2	Chosen to allow for the formation of higher multimeric states.
mini-Lpd monomer-monomer splitting force	10 pN	Used in this study
mini-Lpd monomer-monomer steric repulsion	Radius 30 nm<br>Stiffness 5.0 pN/µm	Chosen empirically to ensure adequate dimerization reactions occur to support the observation of ring structures as determined from a previous study on mini-Lpd monomers[7].

Supplementary Table 2: Table of varied parameters

Parameter	Value	Notes/Reference
**Parameters for simulations with tetravalent crosslinkers (Figure 2, Supplementary Figure 2, Supplementary Figure 4)**		
Binding rates k_{bind}	{10^{-3}, 10^{-2}, 10^{-1}, 10^{0}, 10^{+1}} (1/s)	Range determined based on a previous study with tetramers[5].
Zero-force actin unbinding rates k_{unbind}	{10^{-3}, 10^{-2}, 10^{-1}, 10^{0}, 10^{+1}} (1/s)	
**Parameters for simulations that establish power law relations (Figure 3B, 3D, 6B, 7B)**		
Number of Filaments	{5, 10, 15, 20, 25, 30, 35, 40, 45, 50, 55, 60, 65, 70, 75, 80, 85, 90, 95, 100}	Used in this study
Initial Droplet Diameter	{0.5, 1.0, 1.5, 2.0, 2.5, 3.0, 3.5, 4.0} μm	Used in this study
Surface Tension $\sigma_{surface}$	{2, 4, 8} pN/μm	Used in this study
Multimer formation rate used for power law simulations k_{form}	10 (1/s)	For Fig. 6B. Determined from dynamic multimer simulations.
Multimer splitting rate used for power law simulations k_{split}	0.01 (1/s)	For Fig. 6B. Determined from dynamic multimer simulations.
**Parameters for simulations of capping protein (Figure 4, Supplementary Figure 5)**		
Capping rates k_{cap}	{2^{0}, $2^{+0.5}$, $2^{+1.0}$, $2^{+1.5}$, $2^{+2.0}$, $2^{+2.5}$, $2^{+3.0}$, $2^{+3.5}$} (1/s)	Used in this study
Uncapping rates k_{uncap}	1.0 s^{-1}	Used in this study
**Parameters for simulations of dynamically multimerizing proteins (Figure 6, Supplementary Figure 6)**		
Multimer forming rates k_{form}	{10^{-3}, 10^{-2}, 10^{-1}, 10^{0}, 10^{+1}} (1/s)	Range determined based on a previous study with dynamic multimers[7].
Multimer splitting rates k_{split}	{10^{-3}, 10^{-2}, 10^{-1}, 10^{0}, 10^{+1}} (1/s)	
**Parameters for simulations varying droplet material properties (Figure 7, Supplementary Figure 4, Supplementary Figure 7)**		
Surface Tension $\sigma_{surface}$	{10^{-3}, 10^{-2}, 10^{-1}, 10^{0}, 10^{+1}} (pN/μm)	Used in this study
Effective Viscosity: $\mu_{effective}$	{100, 150, 200, 300, 400} (pN s/μm)	Used in this study

Supplementary Table 3: Table of actin network shapes for each (k_{bind}, k_{unbind}) condition

k_{bind} (1/s) \ k_{unbind} (1/s)	0.001	0.01	0.1	1.0	10.0
10.0	Tight Shell	Loose Shell	10% Loose Shell<br>90% Ring	Ring	Ring
1.0	Tight Shell	90% Loose Shell<br>10% Ring	Ring	Ring	Disc
0.1	80% Tight Shell<br>20% Loose Shell	Loose Shell	Ring	Disc	Disc
0.01	90% Tight Shell<br>10% Loose Shell	20% Ring<br>80% Disc	Disc	Disc	Disc
0.001	Disc	Disc	Disc	Disc	Disc

Supplementary Figures

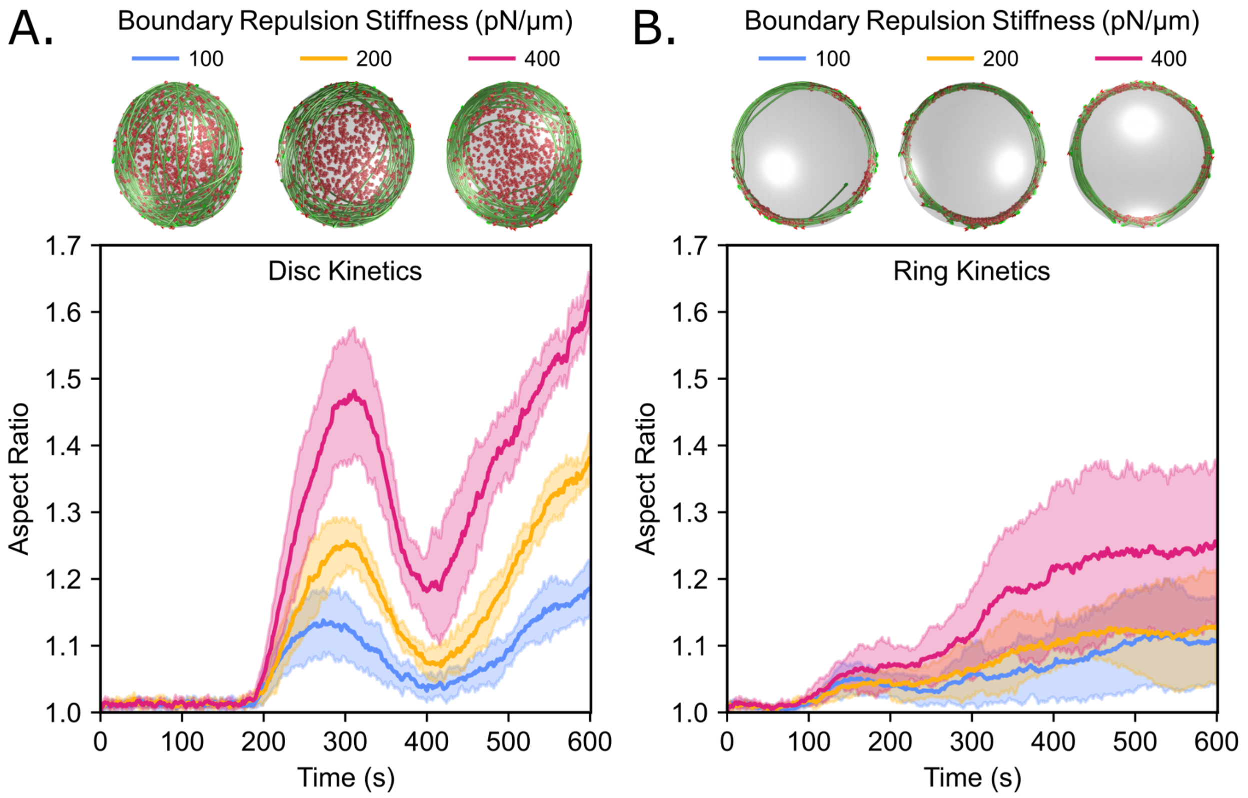

Supplementary Figure 1: Magnitude of condensate aspect ratio varies with filament boundary repulsion stiffness. A) Time series showing the mean (solid line) and standard deviation (shaded area) of droplet aspect ratio for each actin boundary repulsion condition for simulations of VASP that have disc-forming kinetics (k_{bind} = 0.1 s^{-1}, k_{unbind} = 1.0 s^{-1}). **B)** Time series showing the mean (solid line) and standard deviation (shaded area) of droplet aspect ratio for each actin boundary repulsion condition for simulations of VASP that have ring-forming kinetics (k_{bind} = 10.0 s^{-1}, k_{unbind} = 1.0 s^{-1}). Representative final snapshots of each condition are included above each plot. For **A** and **B**, 10 replicates are considered per condition. Source data are provided as a Source Data file.

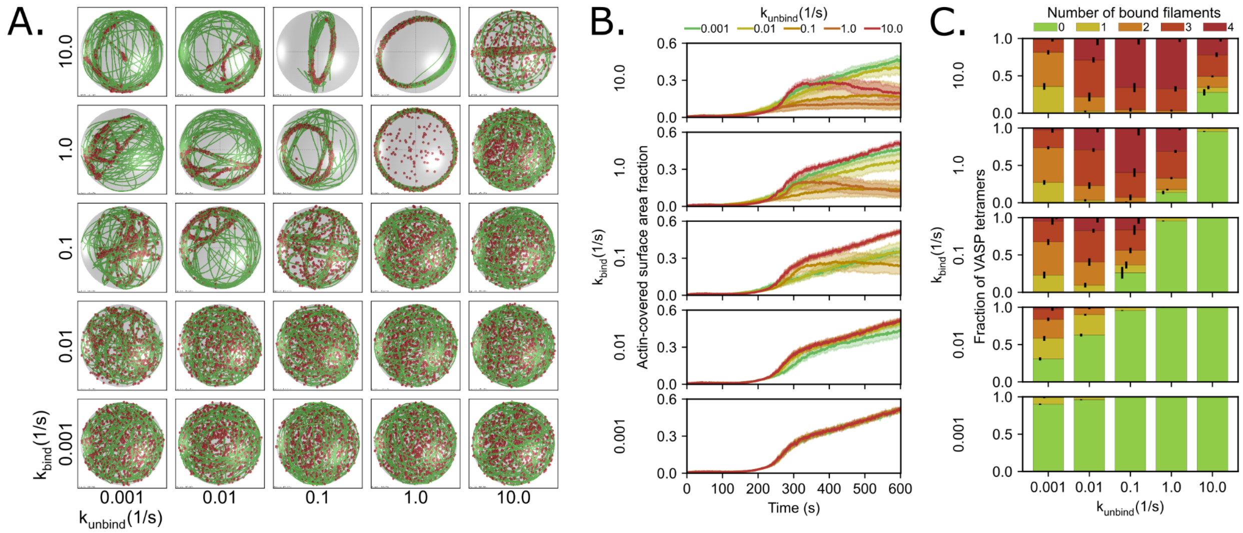

Supplementary Figure 2: Varied crosslinker kinetics in simulations of rigid-boundary spherical droplets. A) Representative final snapshots (t = 600 s) from simulations at various binding and unbinding rates within rigid-boundary spherical droplets (R = 1 μm) containing 30 actin filaments (green) and 1000 tetravalent crosslinkers (red spheres). The binding rates of the tetravalent crosslinkers are varied along each column, and unbinding rates are varied along each row. The polymerization rate at the plus (+) end is constant at 0.0103 μm/s, and neither end undergoes depolymerization. **B)** Actin-covered surface area fraction for varied tetravalent crosslinker binding and unbinding kinetics. **C)** Fraction of tetravalent crosslinkers bound to 0, 1, 2, 3, or 4 actin filaments for each condition. The error bars represent the standard deviation. Data was obtained from the last 30 snapshots (5%) of each replicate. For **B** and **C**, 10 replicates are considered per condition. Source data are provided as a Source Data file.

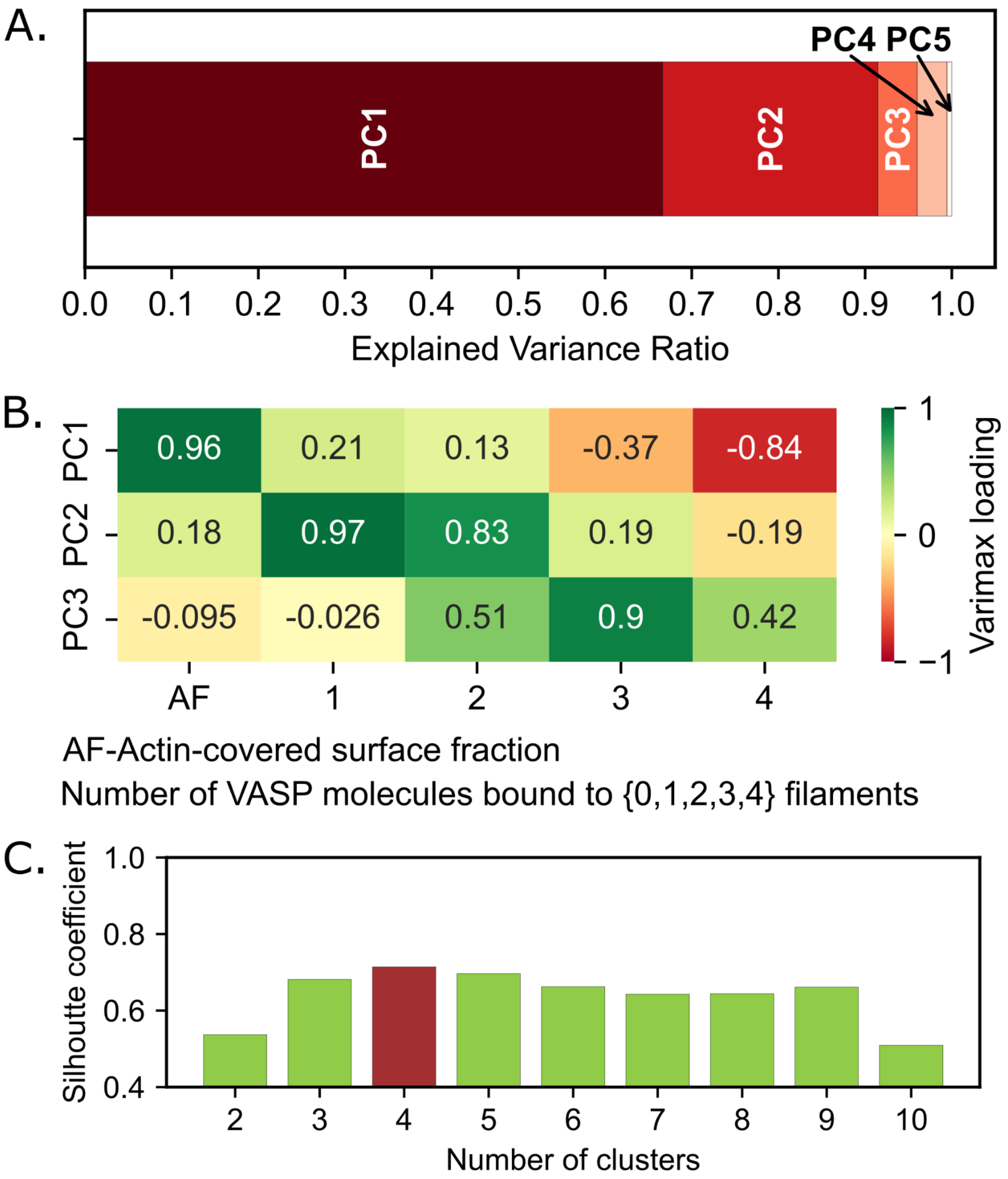

Supplementary Figure 3: K-means clustering and PCA optimization. A) Principal component analysis was employed to find orthogonal axes corresponding to the data represented by the actin-covered surface fraction and the fraction of VASP tetramers bound to 1, 2, 3, and 4 actin filaments. **B)** Varimax loadings were calculated for each of the PCs with the five factors listed above. Loadings reveal that PC1 primarily reflects information positively correlated with the actin-covered surface fraction and negatively correlated with the fraction of VASP tetramers bound to four filaments, PC2 reflects a positive correlation with the fraction of VASP bound to one and two filaments, while PC3 reflects a positive correlation with the fraction of VASP bound to 3 filaments. **C)** Silhouette coefficient shows that the dataset is made up of a maximum of 4 clusters. Data used: Last 5 snapshots from each of the 10 replicates per (k_{bind}, k_{unbind}) pair value. Source data are provided as a Source Data file.

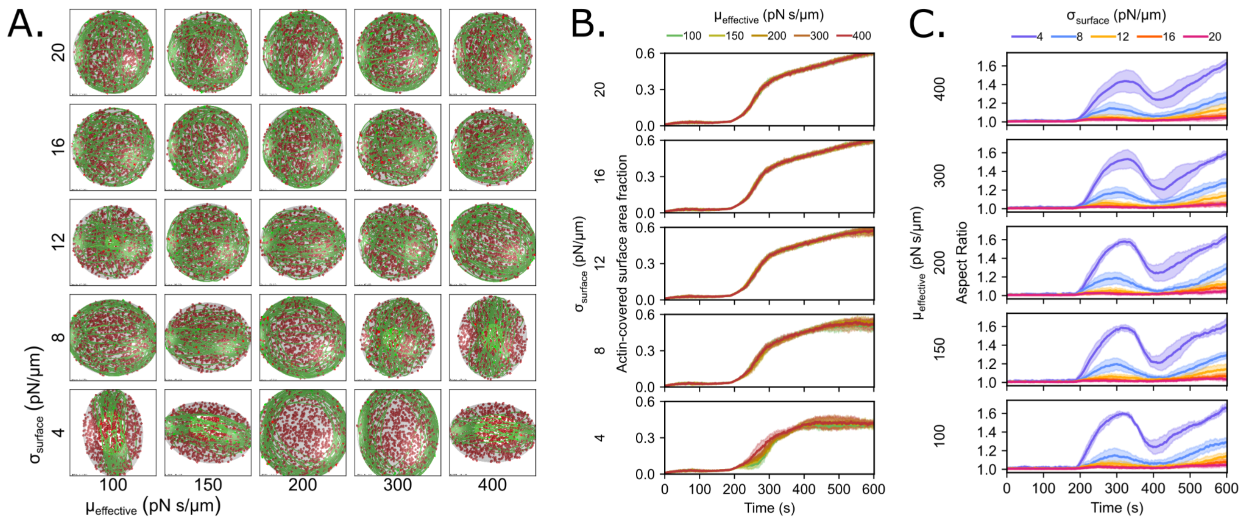

Supplementary Figure 4: Interfacial properties of the droplet surface govern deformation and determine whether the actin network will form shells or discs. A) Representative final snapshots (t = 600 s) from simulations varying the properties of the deformable droplet boundary (initially spherical with R = 1 μm) containing 30 actin filaments (green) and 1000 tetravalent crosslinkers (red spheres) with shell/disc-forming kinetics. The effective viscosity, which attenuates the rate of deformation, is varied along each column, and the surface tension, which describes the innate resistance of the droplet boundary to deformation, is varied along each row. The polymerization rate at the plus (+) end is constant at 0.0103 μm/s, and neither end undergoes depolymerization. **B)** Time series showing the mean (solid line) and standard deviation (shaded area) of the actin-covered surface area fraction for varied droplet interfacial properties. **C)** Time series showing the mean (solid line) and standard deviation (shaded area) of droplet aspect ratio for each condition. Surface tension is varied within each plot with the same effective viscosity. The aspect ratio is defined as the ratio between the longest and shortest axis of the ellipsoid (AR = a/c), where a ≥ b ≥ c. For **B** and **C**, 10 replicates are considered per condition. Source data are provided as a Source Data file.

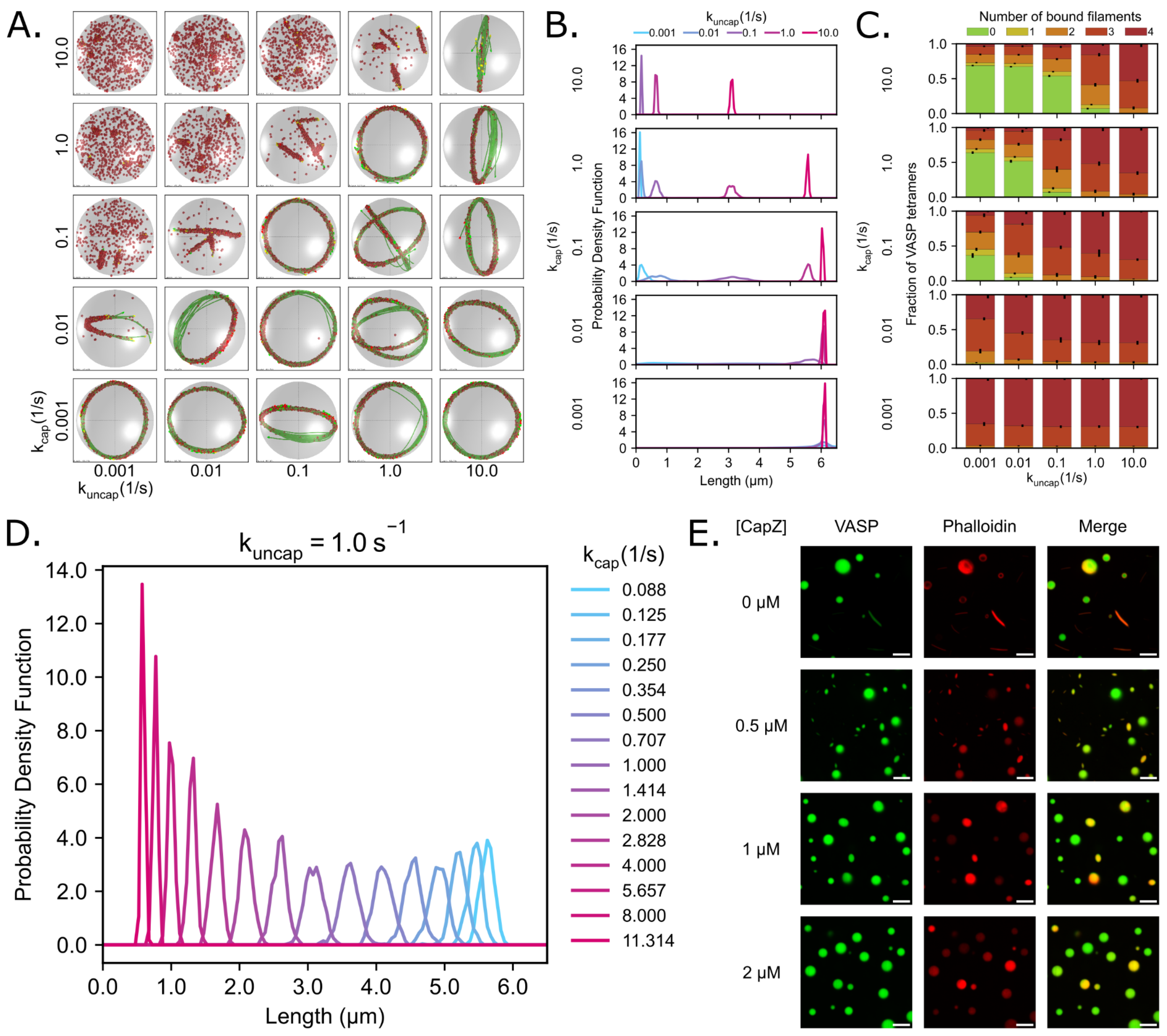

Supplementary Figure 5: Capping and uncapping kinetics of capping protein tune filament length in rigid and deformable droplets. A) Representative final snapshots (t = 600 s) from simulations at various capping and uncapping rates within droplets with a rigid spherical boundary (R = 1 μm) containing 30 actin filaments (green) and 1000 tetravalent crosslinkers (red spheres). The filament growth rate is fixed at 0.0103 μm/s, and the capping rates are varied along each column, and the uncapping rates are varied along each row. The binding rates of the tetravalent crosslinkers are chosen to promote ring formation when $L_{fil} = 2\pi R$. **B)** Probability density function of the final filament length for each simulation condition shown in **A**. As the capping rate increases, the probability of finding longer filaments decreases. The uncapping rate k_{uncap} is changed within each subpanel, and the capping rate k_{cap} is changed between the subpanels. **C)** Fraction of tetravalent crosslinkers bound to 0, 1, 2, 3, or 4 actin filaments for each condition. The error bars represent the standard deviation. Data was obtained from the last 30 snapshots (5%) of each replicate. **D)** Probability density function of the final filament length for an extended set of simulation conditions where capping rates are varied while the uncapping rate is held constant at 1.0 s^{-1} to sample the transition between short rods and ring structures within deformable droplets. The deformable boundary has a surface tension of 4 pN/μm and an effective viscosity of 100 pN

s/μm. As the capping rate increases, the probability of finding longer filaments decreases. Additionally, the probability density function narrows when k_{uncap}/k_{cap} is small or large and is broader when $k_{cap} \approx k_{uncap}$. For **B**, **C**, and **D**, 10 replicates are considered per condition. **E)** Phalloidin-iFluor-594 staining of Atto 488 labeled VASP condensates with 3 μM actin, displaying polymerized actin within the condensates and the disruption of rings with increasing CapZ (not labelled) concentration. Scale bars, 5 μm. Source data are provided as a Source Data file.

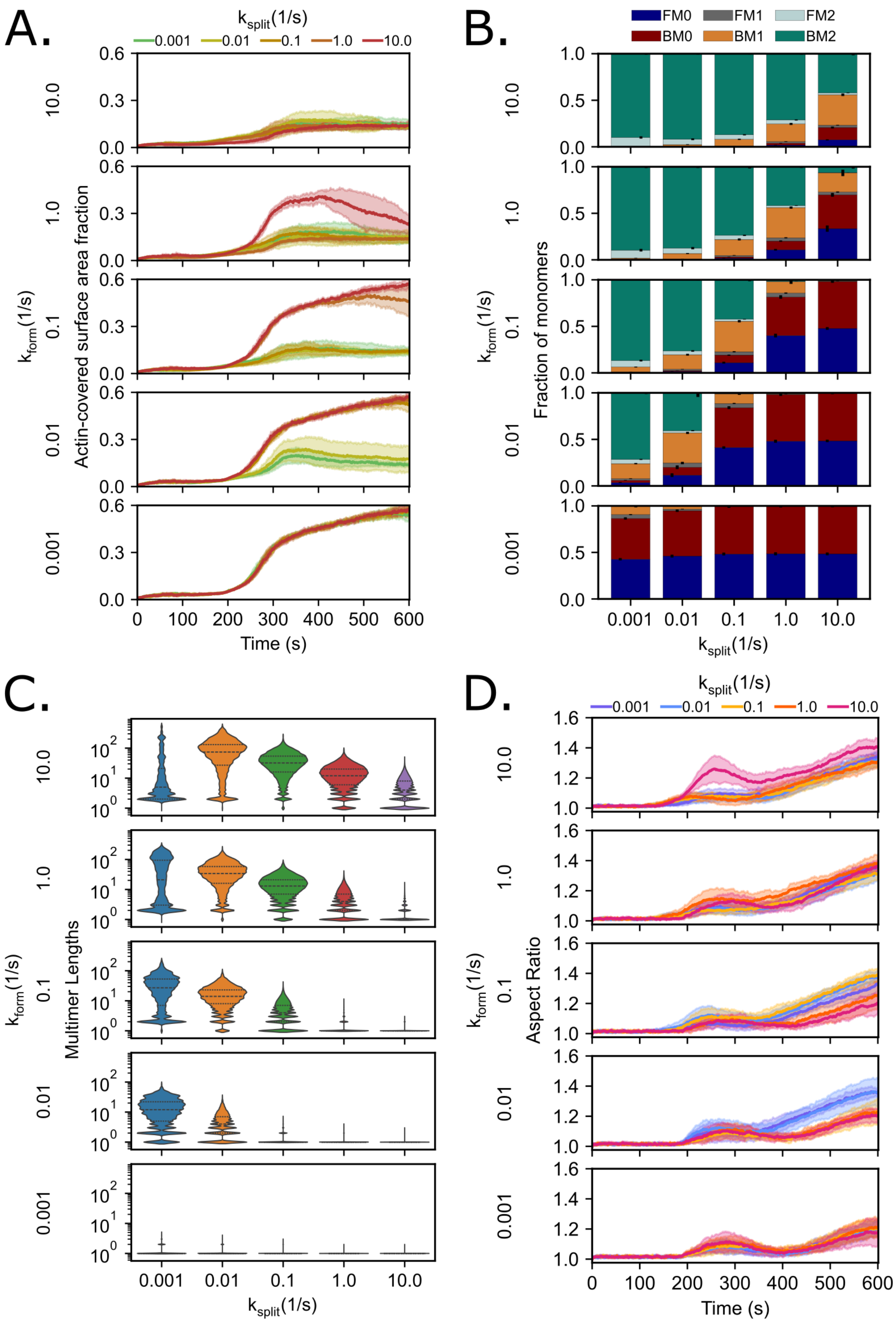

A.
k_{split}(1/s)
0.001
0.01
0.1
1.0
10.0
Actin-covered surface area fraction
k_{form}(1/s)
Time (s)
B.
FM0
FM1
FM2
BM0
BM1
BM2
Fraction of monomers
k_{form}(1/s)
k_{split}(1/s)
C.
Multimer Lengths
k_{form}(1/s)
k_{split}(1/s)
D.
k_{split}(1/s)
Aspect Ratio
k_{form}(1/s)
Time (s)

Supplementary Figure 6: Multivalent droplet environments exhibit disc formation and a power law scaling relationship between the initial diameter of the droplet and the number of filaments. A) Time series showing the mean (solid line) and standard deviation (shaded area) of the actin-covered surface area fraction for varied multimer forming and splitting kinetics. **B)** Fraction of monomers in various multimerization and actin-bound states for each condition. BM (Bound Monomer) refers to monomeric units that are bound to an actin filament, while FM (Free Monomer) refers to those that are not bound to actin; the corresponding number (0, 1, or 2) indicates the number of other monomers that a single monomeric unit is bound to. The error bars represent the standard deviation. Data was obtained from the last 30 snapshots (5%) of each replicate. **C)** Violin plots showing the distribution of multimer lengths for each simulation condition for systems. Multimer lengths are counted as the number of monomers that constitute a single multimer chain. Violin plot densities are normalized such that all plots are fit to the same width. Median (dashed line) and quartiles (dotted lines) are shown. Data was obtained from the last 30 snapshots (5%) of each replicate. **D)** Time series showing the mean (solid line) and standard deviation (shaded area) of droplet aspect ratio for each condition. The aspect ratio is defined as the ratio between the longest and shortest axes of the ellipsoid (AR = a/c), where a ≥ b ≥ c. For **A**, **B**, **C**, and **D**, 10 replicates are considered per condition. Source data are provided as a Source Data file.

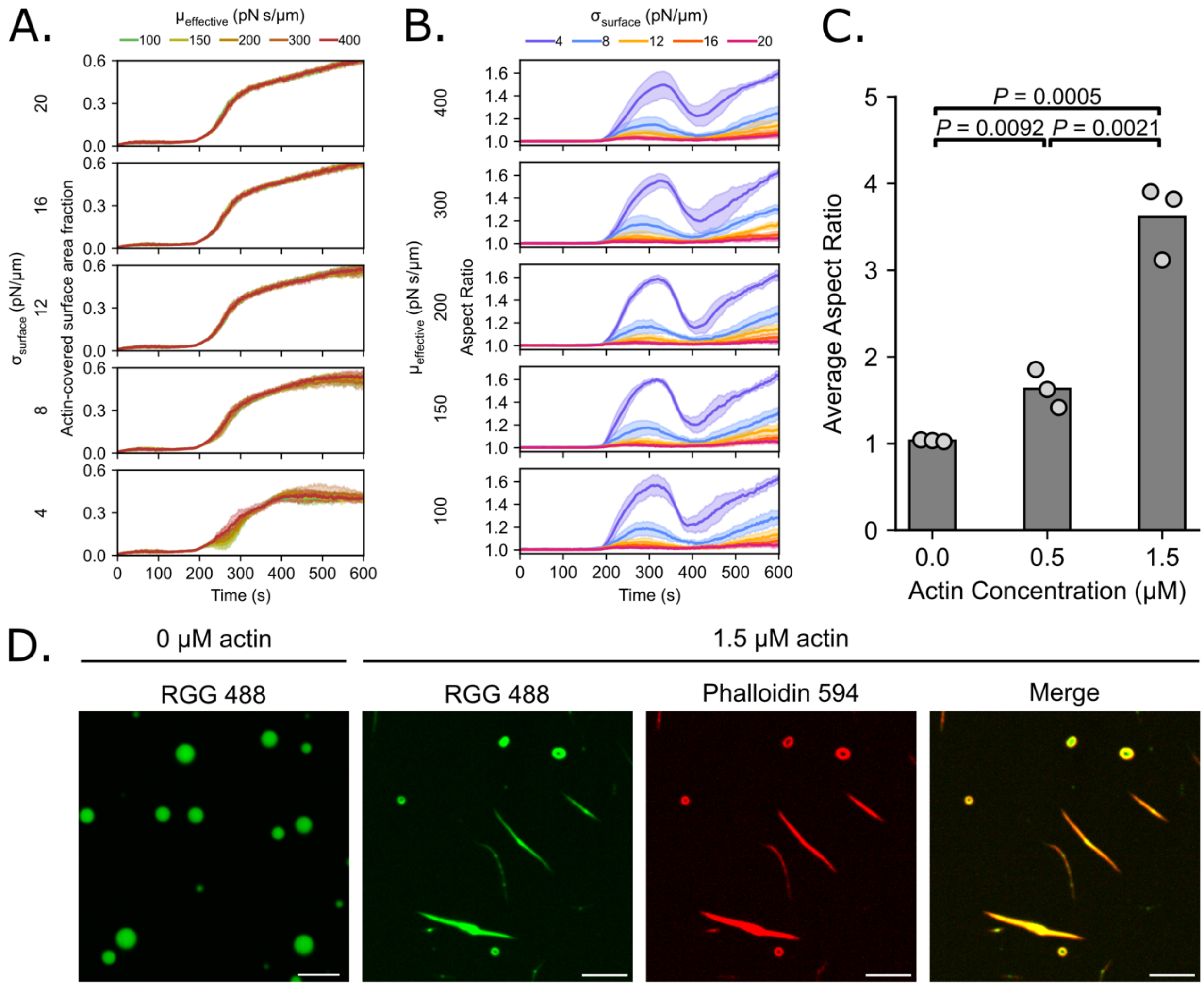

Supplementary Figure 7: Interfacial properties of the droplet surface govern deformation and determine whether the actin network will form shells or discs. A) Each subpanel shows the time series of mean (solid line) and standard deviation (shaded area) of the actin-covered surface area fraction colored by effective viscosity at a given surface tension value mentioned to the left. **B)** Each subpanel shows the time series of mean (solid line) and standard deviation (shaded area) corresponding to droplet aspect ratio colored by surface tension (value shown in legend above). The effective viscosity of the droplet interface is varied between the subpanels and is mentioned to the left. Surface tension is varied within each plot with the same effective viscosity. The aspect ratio is defined as the ratio between the longest and shortest axis of the ellipsoid (AR = a/c), where a ≥ b ≥ c. For **A** and B, 10 replicates are considered per condition. C) Quantification of the average aspect ratio for conditions shown in 7C, showing an increase in aspect ratio with increasing actin concentration. Data are the mean across three independent experiments. The overlaid white circles denote the means of each replicate. Significance values are determined using an unpaired, two-tailed t-test on the means of the replicates, n = 3. Buffer conditions for all conditions were 20 mM Tris pH 7.4, 50 mM NaCl, 5 mM TCEP, and 3% (w/v) PEG 8000. D) Phalloidin-iFluor-594 staining of Atto 488 labeled RGG condensates with 0 μM and 1.5 μM actin, displaying polymerized actin within the condensates and condensate deformation in the presence of actin. Scale bars, 5 μm. Source data are provided as a Source Data file.

References

1. Wang, H., Kelley, F. M., Milovanovic, D., Schuster, B. S. & Shi, Z. Surface tension and viscosity of protein condensates quantified by micropipette aspiration. *Biophys. Rep. (N. Y.)* **1**, 100011 (2021).

2. Dmitrieff, S., Alsina, A., Mathur, A. & Nédélec, F. J. Balance of microtubule stiffness and cortical tension determines the size of blood cells with marginal band across species. *Proc. Natl. Acad. Sci. U. S. A.* **114**, 4418–4423 (2017).

3. Mogilner, A. & Oster, G. Cell motility driven by actin polymerization. *Biophys. J.* **71**, 3030–3045 (1996).

4. Gittes, F., Mickey, B., Nettleton, J. & Howard, J. Flexural rigidity of microtubules and actin filaments measured from thermal fluctuations in shape. *J. Cell Biol.* **120**, 923–934 (1993).

5. Chandrasekaran, A., Graham, K., Stachowiak, J. & Rangamani, P. Kinetic trapping organizes actin filaments within liquid-like protein droplets. *Nature Communications* **15**, 3139 (2024).

6. Ferrer, J. M. *et al.* Measuring molecular rupture forces between single actin filaments and actin-binding proteins. *Proc. Natl. Acad. Sci. U. S. A.* **105**, 9221–9226 (2008).

7. Walker, C. *et al.* Liquid-like condensates that bind actin promote assembly and bundling of actin filaments. *Dev. Cell* (2025) doi:10.1016/j.devcel.2025.01.012.
