# Supplementary material for: Mechanochemical feedback between confinement and actin crosslinking drives the shape dynamics of liquid-like droplets
Source: Nat Commun. 2026 Feb 23;17:3068. doi: 10.1038/s41467-026-69803-4 (PMC13039813; doi:10.1038/s41467-026-69803-4)
Supplement: Supplementary file 2 — Description of Additional Supplementary File [file 41467_2026_69803_MOESM2_ESM.pdf]

Description of Additional Supplementary Files

Supplementary Movie 1: Representative simulation trajectory corresponding to a spherical shell with a nearly flat aspect ratio time series associated with spherical shell formation with aspect ratio near 1.0.

Supplementary Movie 2: Representative simulation trajectory corresponding to a nearly linear monotonic aspect ratio growth into a long rod-like morphology.

Supplementary Movie 3: Representative simulation trajectory corresponding to a nonmonotonic aspect ratio time series associated with disc formation and dynamic snapping.
