# Supplementary material for: Mechanochemical feedback between confinement and actin crosslinking drives the shape dynamics of liquid-like droplets
Source: Nat Commun. 2026 Feb 23;17:3068. doi: 10.1038/s41467-026-69803-4 (PMC13039813; doi:10.1038/s41467-026-69803-4)
Supplement: Supplementary file 6 — Reporting summary [file 41467_2026_69803_MOESM6_ESM.pdf]

nature portfolio

Corresponding author(s): Jeanne Stachowiak, Padmini Rangamani

Last updated by author(s): Jan 26, 2026

Reporting Summary

Nature Portfolio wishes to improve the reproducibility of the work that we publish. This form provides structure for consistency and transparency in reporting. For further information on Nature Portfolio policies, see our Editorial Policies and the Editorial Policy Checklist.

Statistics

For all statistical analyses, confirm that the following items are present in the figure legend, table legend, main text, or Methods section.

n/a	Confirmed	
☐	☒	The exact sample size (*n*) for each experimental group/condition, given as a discrete number and unit of measurement
☐	☒	A statement on whether measurements were taken from distinct samples or whether the same sample was measured repeatedly
☐	☒	The statistical test(s) used AND whether they are one- or two-sided<br>*Only common tests should be described solely by name; describe more complex techniques in the Methods section.*
☒	☐	A description of all covariates tested
☒	☐	A description of any assumptions or corrections, such as tests of normality and adjustment for multiple comparisons
☐	☒	A full description of the statistical parameters including central tendency (e.g. means) or other basic estimates (e.g. regression coefficient) AND variation (e.g. standard deviation) or associated estimates of uncertainty (e.g. confidence intervals)
☐	☒	For null hypothesis testing, the test statistic (e.g. *F*, *t*, *r*) with confidence intervals, effect sizes, degrees of freedom and *P* value noted<br>*Give P values as exact values whenever suitable.*
☒	☐	For Bayesian analysis, information on the choice of priors and Markov chain Monte Carlo settings
☒	☐	For hierarchical and complex designs, identification of the appropriate level for tests and full reporting of outcomes
☒	☐	Estimates of effect sizes (e.g. Cohen's *d*, Pearson's *r*), indicating how they were calculated

Our web collection on statistics for biologists contains articles on many of the points above.

Software and code

Policy information about availability of computer code

Data collection	All microscopy data was collected on a spinning disc operating on Olympus cellSens Dimension software v3.2. The customized Cytosim source code used to generate trajectories and the Python scripts used to analyze the Cytosim trajectories have been deposited in a Zenodo repository (https://doi.org/10.5281/zenodo.18373603). The code is also available on GitHub (https://github.com/RangamaniLabUCSD/abp-deformable-droplet-Mansour-2025.git).
Data analysis	The Python scripts used to analyze the Cytosim trajectories, Cytosim input files, and data generated in this study from experiments and simulations have been deposited in a Zenodo repository (https://doi.org/10.5281/zenodo.18373603). The code and data generated in this study is also available on GitHub (https://github.com/RangamaniLabUCSD/abp-deformable-droplet-Mansour-2025.git). All data analysis for simulations were performed on Python 3.11.5.

For manuscripts utilizing custom algorithms or software that are central to the research but not yet described in published literature, software must be made available to editors and reviewers. We strongly encourage code deposition in a community repository (e.g. GitHub). See the Nature Portfolio guidelines for submitting code & software for further information.

Data

Policy information about availability of data

All manuscripts must include a data availability statement. This statement should provide the following information, where applicable:

- Accession codes, unique identifiers, or web links for publicly available datasets
- A description of any restrictions on data availability
- For clinical datasets or third party data, please ensure that the statement adheres to our policy

The Cytosim input files and data generated in this study from experiments and simulations have been deposited in a Zenodo repository (https://doi.org/10.5281/zenodo.18373603). The data generated in this study is also available on GitHub (https://github.com/RangamaniLabUCSD/abp-deformable-droplet-Mansour-2025.git). Source data are provided with this paper.

Research involving human participants, their data, or biological material

Policy information about studies with human participants or human data. See also policy information about sex, gender (identity/presentation), and sexual orientation and race, ethnicity and racism.

Reporting on sex and gender	Not applicable as human participants are not a part of this study.
Reporting on race, ethnicity, or other socially relevant groupings	Not applicable as human participants are not a part of this study.
Population characteristics	Not applicable as human participants are not a part of this study.
Recruitment	Not applicable as human participants are not a part of this study.
Ethics oversight	Not applicable as human participants are not a part of this study.

Note that full information on the approval of the study protocol must also be provided in the manuscript.

Field-specific reporting

Please select the one below that is the best fit for your research. If you are not sure, read the appropriate sections before making your selection.

☒ Life sciences ☐ Behavioural & social sciences ☐ Ecological, evolutionary & environmental sciences

For a reference copy of the document with all sections, see nature.com/documents/nr-reporting-summary-flat.pdf

Life sciences study design

All studies must disclose on these points even when the disclosure is negative.

Sample size	Sample sizes were chosen to yield sufficient data to model the trends and variability therein, and were also based on our previous experience (Graham, K. et al. Nat. Phys. 19, 574–585 (2023), Chandrasekaran, A. et al. Nat. Commun. 15, 3139 (2024), Walker, C. et al. Dev. Cell (2025)).
Data exclusions	For power law fitting of simulation data, initial diameter values with fewer than 10 samples were excluded to limit the influence of sparsely sampled regions of the parameter space. This criterion was applied uniformly and affected 2 of 12 fits, from which only 2-3 data points per fit were excluded. All other fitting and analyses were performed using the full datasets.
Replication	All experiments were successfully performed and replicated at least 3 times using different stocks of protein to ensure biological independence and reproducibility. All simulations were replicated 10 times.
Randomization	Microscopy images were randomly acquired throughout the sample to avoid bias in data collection. Otherwise, randomization was not applicable as there were no assignments to experimental groups. Stochastic simulations were performed in Cytosim with random seed generators.
Blinding	Blinding was not applicable, as no clinical or animal studies were performed. Data were quantified according to objective parameters as described in the methods. Microscopy images were taken randomly throughout the sample to avoid bias in data collection.

Reporting for specific materials, systems and methods

We require information from authors about some types of materials, experimental systems and methods used in many studies. Here, indicate whether each material, system or method listed is relevant to your study. If you are not sure if a list item applies to your research, read the appropriate section before selecting a response.

Materials & experimental systems

n/a	Involved in the study
☒	☐ Antibodies
☒	☐ Eukaryotic cell lines
☒	☐ Palaeontology and archaeology
☒	☐ Animals and other organisms
☒	☐ Clinical data
☒	☐ Dual use research of concern
☒	☐ Plants

Methods

n/a	Involved in the study
☒	☐ ChIP-seq
☒	☐ Flow cytometry
☒	☐ MRI-based neuroimaging

Plants

Seed stocks

Report on the source of all seed stocks or other plant material used. If applicable, state the seed stock centre and catalogue number. If plant specimens were collected from the field, describe the collection location, date and sampling procedures.

Novel plant genotypes

Describe the methods by which all novel plant genotypes were produced. This includes those generated by transgenic approaches, gene editing, chemical/radiation-based mutagenesis and hybridization. For transgenic lines, describe the transformation method, the number of independent lines analyzed and the generation upon which experiments were performed. For gene-edited lines, describe the editor used, the endogenous sequence targeted for editing, the targeting guide RNA sequence (if applicable) and how the editor was applied.

Authentication

Describe any authentication procedures for each seed stock used or novel genotype generated. Describe any experiments used to assess the effect of a mutation and, where applicable, how potential secondary effects (e.g. second site T-DNA insertions, mosiacism, off-target gene editing) were examined.
